# *Candida albicans* scleral abscess in a HIV-positive patient and its successful resolution with antifungal therapy—a first case report

**DOI:** 10.1186/s12348-016-0092-1

**Published:** 2016-06-22

**Authors:** Hitesh Sharma, Sridharan Sudharshan, Lily Therese, Mamta Agarwal, Jyotirmay Biswas

**Affiliations:** Medical Research Foundation, Sankara Nethralaya, 18, College Road, Chennai, 600 006 Tamil Nadu India; Uveitis and Ocular Pathology Department, Sankara Nethralaya, 18, College Road, Nungambakkam, Chennai 600 006 India

**Keywords:** Scleral abscess, Fungal, *Candida albicans*, Antifungal therapy, HIV-positive patient

## Abstract

**Background:**

Fungal infection of the sclera is very rare. No case of fungal scleral abscess in a HIV-positive patient has been reported. We report a case of scleral abscess caused by *Candida albicans* and its successful resolution following antifungal therapy in a HIV-positive patient.

**Findings:**

A 57-year-old diabetic Asian (Indian) who was on highly active antiretroviral therapy for the last 10 years presented with 2 weeks’ history of redness in his right eye. Examination revealed localised scleral inflammation with central ulceration in the inferior quadrant of the right eye. Initially, the ulcer scrapings revealed no microbial organism. Progression of ulcer although on empirical antibiotic therapy required repeat scrapings which showed *Candida albicans* species in culture sensitive to amphotericin and natamycin. Aggressive topical and systemic antifungals resulted in dramatic and complete healing of the ulcer in 3 weeks. Vision was well maintained at 20/30 throughout the treatment course, and the fundus remained normal.

**Conclusions:**

This is the first ever case of fungal scleral abscess in a HIV patient to be reported emphasizing there is a need for high vigilance to suspect an infective aetiology of scleritis in patients with immunocompromised status. Prompt microbial assessment and appropriate antifungals can decrease morbidity in these unusual but serious cases as illustrated in this case.

## Findings

### Introduction

Infections are uncommon causes of scleral inflammation [1]. Diagnosis is often difficult and gets delayed as the clinical picture appears similar to the more common cause; the immune-mediated disease. Fungal infections of the sclera have been reported following surgeries for retinal detachment [2–5], pterygium [6, 7], cataract [8, 9] and as a part of systemic fungal infections [10, 11]. All these reports are in immunocompetent individuals with a significant inflammatory response.

Acquired immune deficiency syndrome (AIDS) patients are prone to many opportunistic fungal infections, but ocular fungal infections are rare and usually do not involve the sclera [12]. We report a case of fungal scleral abscess caused by *Candida albicans* in a patient with AIDS and its successful resolution following antifungal therapy.

### Case report

A 57-year-old Asian (Indian) male was first seen at our hospital in July 2015. He came to us with a history of redness in his right eye for 15 days associated with pain and watering. He had no complaints regarding his vision. He was a known diabetic for 20 years and was detected to be infected with human immunodeficiency virus (HIV) 10 years back. His CD4 count was 461cells/mm^3^ and was on highly active antiretroviral therapy (HAART). His blood sugar levels were moderately controlled. There was no history of trauma or any other significant history.

**Fig. 1:**
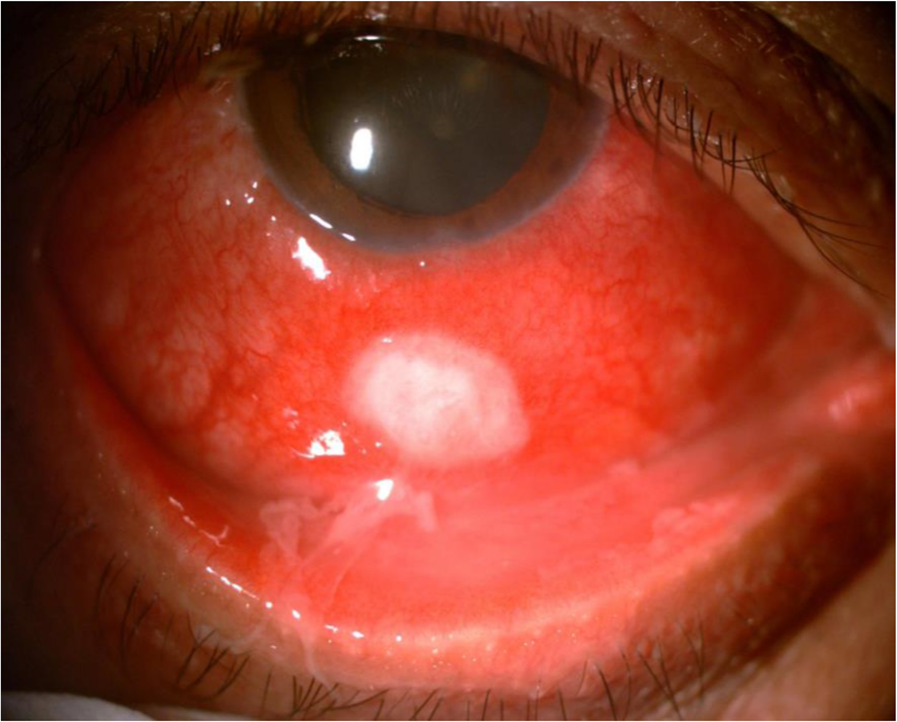
Slit lamp photograph showing the scleral abscess

On examination, his best-corrected visual acuity (BCVA) was 6/9, N6 in both eyes. Slit lamp examination of the right eye revealed evidence of conjunctival congestion and an oval scleral ulcerative lesion around 3 mm posterior from the corneal limbus at around 6 o’clock meridian, measuring 6 mm by 4.5 mm (Fig. 1). The ulcer had dense infiltrates with a pseudomembrane. The cornea was clear, and the anterior chamber was deep and quiet. Posterior segment examination showed a clear vitreous and a normal fundus. Left eye examination was within normal limits. Intraocular pressure by applanation tonometry was 14 mmHg in both eyes. On systemic examination, there was no *Candida* or any fungal infection elsewhere in the body.

He was diagnosed as an infective scleral abscess of the right eye. Scraping of the lesion was done and was put on empirical treatment of oral indomethacin and topical moxifloxacin eye drops one hourly.

**Fig. 2:**
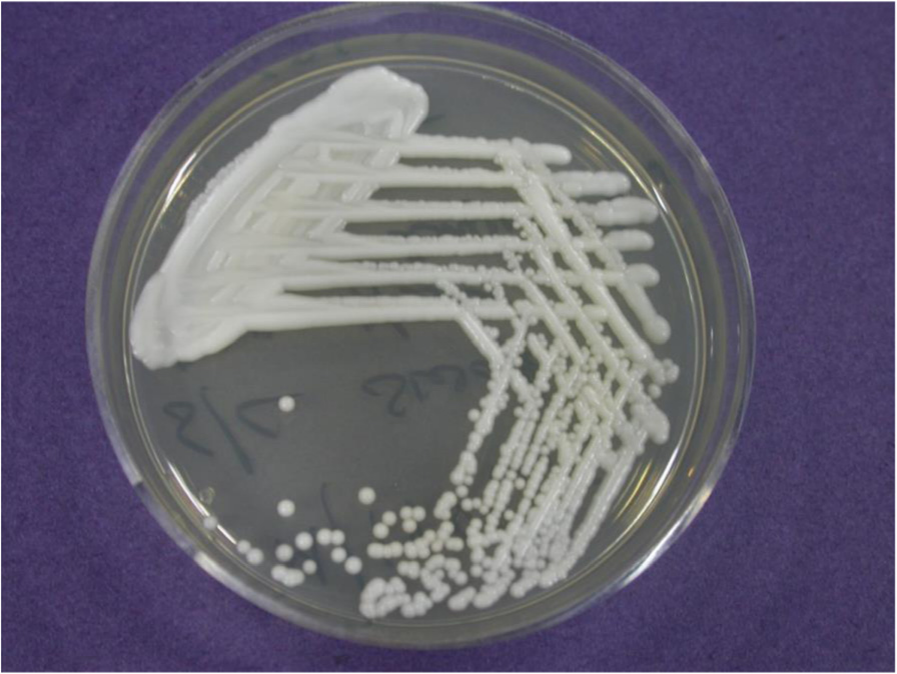
Sabouraud dextrose agar (SDA) showing the isolates of *Candida albicans*

Scraping did not reveal any fungal or bacterial elements, apart from occasional pus cells. He was advised to continue medications. However, after 3 days, culture on Sabouraud dextrose agar showed *Candida albicans* species (Fig. 2.) which confirmed the clinical suspicion. He was given amphotericin B 0.25 % eyedrops one hourly, voriconazole 1 % eyedrops one hourly and ciprofloxacin 0.3 % eyedrops four times per day along with oral voriconazole 200 mg tablet twice daily. The patient was continued on the same medication, while the lesion started increasing in size.

Two days later, the scleral abscess showed signs of improvement. The epithelial defect and congestion persisted. Culture and sensitivity were done which revealed that the fungus is sensitive to amphotericin B and natamycin and resistant to voriconazole, fluconazole and itraconazole. Hence, topical natamycin was added and voriconazole was stopped. The rest of the medications were continued as earlier. The lesion started regressing, and the patient was reviewed after 2 weeks.

**Fig. 3:**
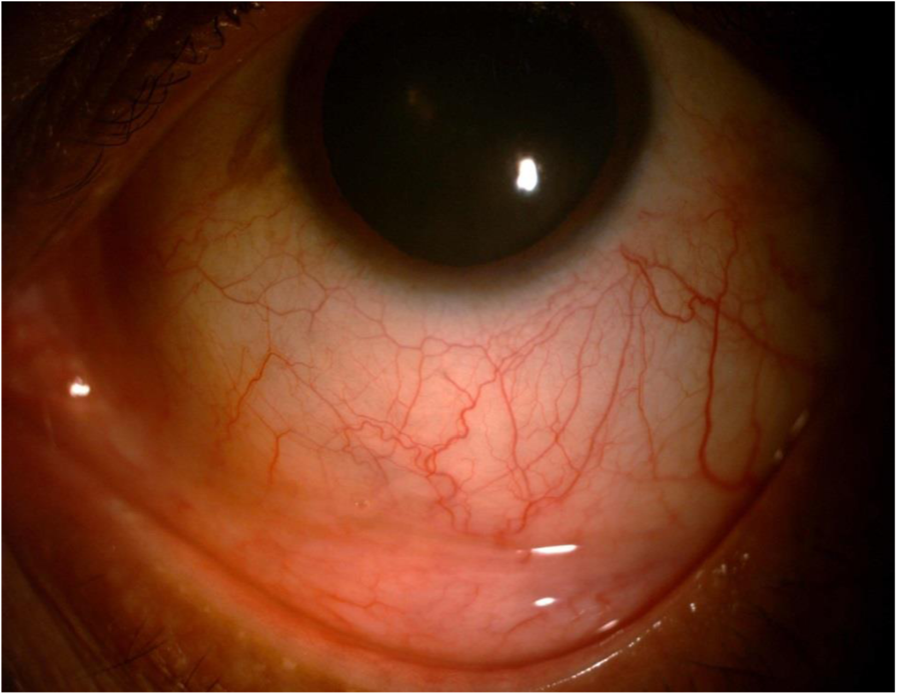
Slit lamp photograph showing completely resolved scleral lesion

At 2-week follow-up, his BCVA was 6/9, N6 in both eyes. The patient had no fresh complaints. Slit lamp examination revealed complete healing of the lesion (Fig. 3). He was advised to stop topical and oral medications and was continued on only topical lubricants. At 1 month of follow-up, there was complete resolution of the lesion. His viral load was increasing along with decrease in CD4 counts and was shifted to second line HAART. At his last visit in January 2016, his viral load was less than 150 copies/ml and was doing well on second line HAART.

### Discussion

Fungal infections of the sclera are devastating cause of infectious scleritis as they are difficult to diagnose and often diagnosed late. The reported incidence of fungal scleritis is around 11 to 38 % of the total infectious causes of scleritis [13–15].

*Candida albicans* is a dimorphic commensal fungus. Candidiasis is usually seen in immunocompromised individuals like HIV-infected patients. Candidiasis has a varied presentation. *Candida albicans* usually causes keratitis, chorioretinitis and endogenous endophthalmitis in HIV/AIDS patients [16–18]. Scleral infection by *Candida albicans* is very rare. Ahn et al. have reported two cases of fungal scleral infection in immunocompetent individuals [19]. Garelick et al. have described a case of *Cryptococcus albidus* in a patient with AIDS [20].

No case of scleral abscess has been reported in any patient with HIV/AIDS. Our patient is a HIV-positive patient and has a scleral abscess caused by *Candida albicans*. Hence, it should be considered as a possible diagnosis and early investigation and treatment should be done, as it can lead to devastating complication like endophthalmitis. Our case also highlights the fact that a strong degree of clinical suspicion backed by appropriate anti-infective (anti-fungal) therapy is a must for the complete resolution of the lesion.

Our case demonstrates the utility of culture and sensitivity in choosing the appropriate anti-fungal agent, since the initial use of a broad spectrum anti-fungal did not yield the required result. Based on culture and sensitivity, specific drugs were used which lead to the complete resolution of lesions.

### Conclusion

In conclusion, we report an uncommon presentation of a *Candida albicans* scleral abscess in an AIDS patient, who was treated promptly by appropriate topical and oral antifungals. Proper scraping and culture and sensitive reporting are an essential component of diagnosis and treating such a rare case thus preventing grave consequences.

### Consent

Written informed consent was obtained from the patient.

### Abbreviations

AIDS, acquired immune deficiency syndrome; HIV, human immunodeficiency virus; HAART, highly active antiretroviral therapy; BCVA, best-corrected visual acuity
